# A single-electrode electrochemical system for multiplex electrochemiluminescence analysis based on a resistance induced potential difference†
†Electronic supplementary information (ESI) available. See DOI: 10.1039/c8sc00410b


**DOI:** 10.1039/c8sc00410b

**Published:** 2018-03-19

**Authors:** Wenyue Gao, Kateryna Muzyka, Xiangui Ma, Baohua Lou, Guobao Xu

**Affiliations:** a State Key Laboratory of Electroanalytical Chemistry , Changchun Institute of Applied Chemistry , Chinese Academy of Sciences , Changchun , Jilin 130022 , P. R. China . Email: guobaoxu@ciac.ac.cn; b University of Chinese Academy of Sciences , Beijing , 100039 , P. R. China; c Laboratory of Analytical Optochemotronics , Department of Biomedical Engineering , Kharkiv National University of Radio Electronics , Kharkiv 61166 , Ukraine

## Abstract

Developing low-cost and simple electrochemical systems is becoming increasingly important but still challenged for multiplex experiments. Here we report a single-electrode electrochemical system (SEES) using only one electrode not only for a single experiment but also for multiplex experiments based on a resistance induced potential difference. SEESs for a single experiment and multiplex experiments are fabricated by attaching a self-adhesive label with a hole and multiple holes onto an ITO electrode, respectively. This enables multiplex electrochemiluminescence analysis with high sensitivity at a very low safe voltage using a smartphone as a detector. For the multiplex analysis, the SEES using a single electrode is much simpler, cheaper and more user-friendly than conventional electrochemical systems and bipolar electrochemical systems using electrode arrays. Moreover, SEESs are free from the electrochemiluminescent background problem from driving electrodes in bipolar electrochemical systems. Since numerous electrodes and cover materials can be used to fabricate SEESs readily and electrochemistry is being extensively used, SEESs are very promising for broad applications, such as drug screening and high throughput analysis.

## Abstract 2

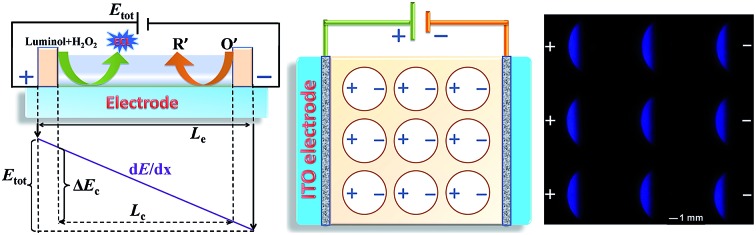
A single-electrode electrochemical system uses only one electrode for multiplex experiments, and is a highly cheap platform for high throughput analysis.

## Introduction

Electrochemistry deals with the interaction between chemical changes and electrical energy transfer.^1^ As an environmentally benign and versatile technique, it has been extensively investigated and widely used in many fields ranging from electroanalysis, energy conversion and storage, electrosynthesis, light-emitting devices, anticorrosion, electroforming, and water splitting to wastewater treatment.^2^–^6^ Traditional electrochemical systems generally employ two electrodes (a working electrode and a counter electrode) or three electrodes (a working electrode, a counter electrode, and a reference electrode) for a single electrochemical experiment.^7^ In recent years, bipolar electrochemistry has attracted much attention in electrochemistry.^8^–^12^ Different from traditional electrochemical systems, bipolar electrochemical systems exert potential control over electrolyte solutions with driving electrodes and redox reactions occur at the two opposite poles of the bipolar electrode (BPE).^13^ This enables simultaneous control of many bipolar electrode arrays with only two driving electrodes for multiplex experiments.^14^–^16^ However, both traditional electrochemical systems and bipolar electrochemical systems require the use of electrode arrays for multiplex experiments. It is time-consuming and expensive to make electrode arrays and/or electrode connectors, particularly electrode arrays with many electrodes. Thus, new low-cost and simple electrochemical systems are required to overcome these limitations.

Electrochemiluminescence (also called electrogenerated chemiluminescence, abbreviated as ECL) is an electrochemical process in which molecules undergo high-energy electron transfer reactions at electrode surfaces to form excited states that emit light.^17^–^19^ As a powerful analytical technique, ECL has been widely used in various assays and shows many advantages, such as rapidity, simplicity, and high sensitivity.^20^–^25^ Since ECL is intrinsically a combination of electrochemistry and spectroscopy, the development of ECL detection methods and the miniaturization of ECL devices highly depend on the development of electrochemical systems.

**Fig. 1:**
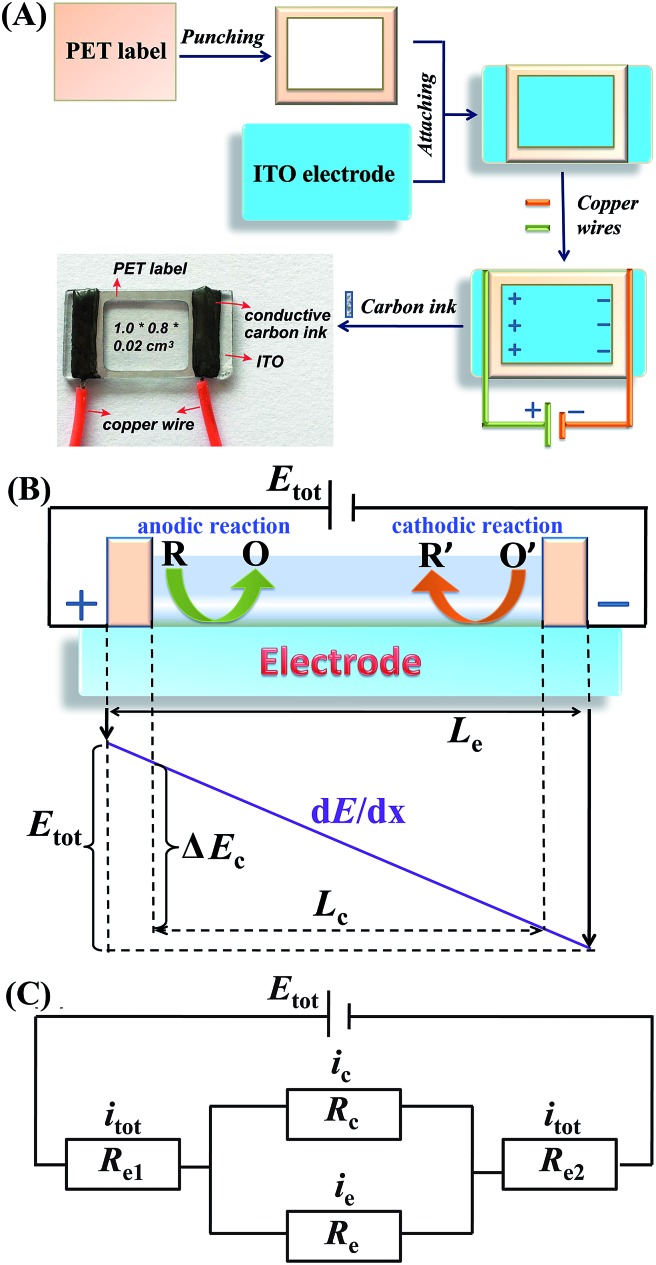
(A) Fabrication procedure of the SEES and the schematic diagram of the SEES. (B) The principle of the SEES. (C) Schematic of the equivalent electrical circuit of the SEES.

In the present study, a single-electrode electrochemical system (SEES) has been developed for the first time and its application in ECL detection has been demonstrated. It is noteworthy that the SEES still uses one electrode not only for single analysis but also for multiplex ECL analysis. As shown in Fig. 1A, the SEES consists of only one electrode in total and an insulating self-adhesive plastic film with a hole to construct a microelectrochemical cell. When an external voltage is applied to both the ends of the single electrode, a potential difference between the two ends of the microelectrochemical cell is generated, thus leading to faradaic reactions. For the multiplex ECL analysis, an insulating self-adhesive plastic film with a series of holes is used to construct a microelectrochemical cell array. In contrast to traditional electrochemical systems and bipolar electrochemical systems which use electrode arrays for high-throughput electrochemical experiments, our SEES uses only one electrode for high-throughput electrochemical experiments and eliminates the need for the complex and costly fabrication of electrode arrays and connectors.

## Results and discussion

### Fabrication and principle of the SEES

The procedure for the fabrication of the SEES and the schematic diagram of the SEES are shown in Fig. 1A. Since indium tin oxide (ITO) with different resistances and polyethylene terephthalate (PET) labels with different sizes are commercially available, we attached a PET label with a rectangular hole onto an ITO electrode to make the SEES. The hole of the PET label was used to form a microelectrochemical cell. The principle and an analogous electrical circuit of the SEES are illustrated in Fig. 1B and C, respectively. When a solution is added to the microelectrochemical cell and an external potential (*E*_tot_) is applied to the opposite ends of the ITO electrode, electric currents are produced between the two ends of the electrode and a potential gradient (d*E*/d*x*) along the electrode is generated due to the resistance of the electrode.^26^ The total current (*i*_tot_) can be carried through both the electrode (*i*_e_) and the solution in the microelectrochemical cell (*i*_c_).^9^ The relative fraction of the current passing through the solution and the electrode depends on the relative resistance values of the solution in the microelectrochemical cell (*R*_c_) and the ITO electrode at the bottom of the microelectrochemical cell (*R*_e_), as given in eqn (1). Fig. S1† shows current–time profiles in the absence and presence of a typical ECL solution, 0.1 M pH 11.0 carbonate buffer containing 10 μM of luminol and 0.1 mM of H_2_O_2_. The addition of the solution has little effect on total currents, indicating that most current passes through the ITO film and *R*_c_ is much larger than *R*_e_. When *R*_c_ is much larger than *R*_e_, most current passes through the ITO film, and the electric field in the electrochemical cell is approximately uniform. The potential difference between the two ends of the microelectrochemical cell (Δ*E*_c_) is the fraction of *E*_tot_ dropped along the length of the microelectrochemical cell (*L*_c_) and mainly depends on the proportion between the length of the microelectrochemical cell and the length of the self-adhesive plastic film on the electrode (*L*_e_) (eqn (2)). If Δ*E*_c_ is large enough, then electrochemical processes will occur simultaneously on the electrode surface at both ends of the microelectrochemical cell.1
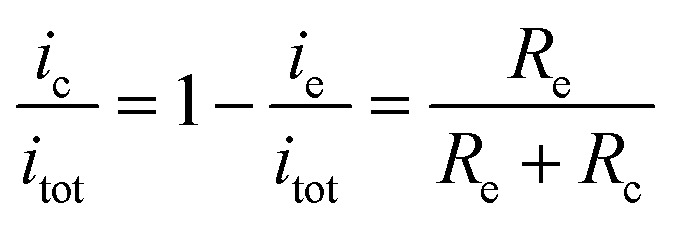

2
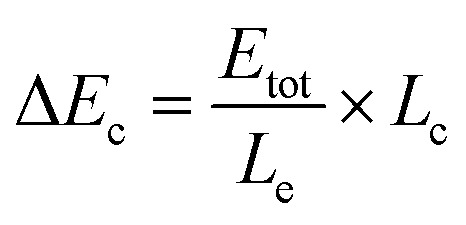



### SEESs for ECL applications

**Fig. 2:**
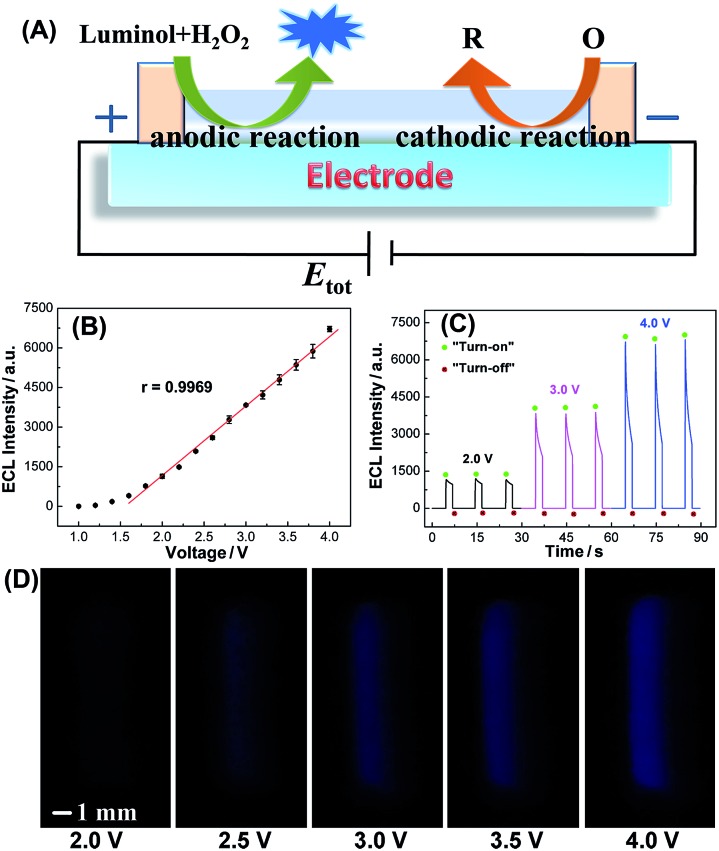
(A) ECL mechanism of a SEES. (B) Dependence of the ECL intensity on the applied external voltage. (C) ECL profiles of three consecutive measurements using a SEES at 2.0 V, 3.0 V and 4.0 V, respectively. The photomultiplier tube (PMT) voltage was set at 400 V. “Turn-on” means that the external power source is turned on to induce ECL; “Turn-off” means that the external power source is turned off. (D) ECL emission images taken using a smartphone at different external voltages. 0.1 M carbonate buffer, pH 11.0; *c* (luminol), 1.0 mM; *c* (H_2_O_2_), 1.0 mM.

ECL is the light emission from the excited states of ECL luminophores produced on the electrode surface *via* an electrochemical reaction.^27^ On the one hand, ECL can serve as an indirect reporter to monitor electrochemical reactions in SEESs that are difficult to be measured by other methods.^26^ On the other hand, SEESs can greatly facilitate multiplex ECL analysis. Therefore, we utilize ECL to demonstrate the feasibility of SEESs for electrochemical applications. Fig. 2A shows the ECL mechanism of a SEES. Luminol and hydrogen peroxide are oxidized on the surface of ITO at the anode and hydrogen peroxide and dissolved oxygen may be reduced on the surface of ITO at the cathode, leading to the generation of ECL on the surface of ITO at the anode. Fig. 2B shows the dependence of the ECL intensity of luminol/H_2_O_2_ on the applied external voltage. As the voltages increase, the ECL signals increase rapidly. When the voltages are higher than 1.6 V, the ECL intensities measured using a PMT-based detector increase linearly with the applied voltage. The ECL intensity increase is due to both the extension of the ECL-emitting region and the local ECL intensity as shown in Fig. 2D.^28^,^29^ The ECL profiles of three consecutive measurements at different external voltages are shown in Fig. 2C. It indicates that the SEES has satisfactory reproducibility. Moreover, intense ECL is clearly observed using a smartphone at the left edge of the microelectrochemical cell on the ITO electrode at a low applied voltage (Fig. 2D), which demonstrates its nice feasibility and attractive potential in visual detection.

Moreover, the effect of ITO resistance on the performance of the SEES is presented in Fig. S2.† The ECL becomes stronger as the ITO resistances increase and is the strongest at an ITO resistance of 100 ohm per square. The change in the current intensity and transparency of ITO contributes to the dependence of ECL. Moreover, ITO with a resistance of 100 ohm per square is cheaper, and thus the SEESs used in the following experiments were all made with ITO of 100 ohm per square.

The dependence of the ECL intensities on the concentrations of luminol is shown in Fig. S3.† The ECL intensity measured using a PMT-based detector has a good linear relationship with the concentrations of luminol from 2.0 nM to 20.0 μM with a correlation coefficient (*r*) of 0.9993. The linear equation is *I* = 213.27 + 1.60*c*, where *I* represents the ECL intensity and *c* is the concentration of luminol in nanomolar. The limit of detection (LOD) is 1.36 nM at a signal-to-noise ratio of 3, which is comparable with that of the most sensitive method reported before.^30^

ECL detection toward hydrogen peroxide using the SEES was investigated to demonstrate its feasibility, simplicity and good sensitivity in electrochemical detection. A low voltage of 2.0 V was applied to the SEES to initiate ECL. As shown in Fig. S4,† the ECL intensities measured using a PMT-based detector increase with the increase in the concentrations of H_2_O_2_ from 1.0 μM to 1.0 mM and then level off. Since the concentration of the buffer used is one hundred times larger than the maximum H_2_O_2_ concentrations detected, the change of H_2_O_2_ concentrations has little effect on solution resistance, and thus the change of solution resistance resulting from the change of H_2_O_2_ concentrations has a negligible effect on ECL. To confirm this, we have measured ECL intensities at different buffer concentrations. The ECL intensity keeps essentially constant even when the buffer concentrations change from 50 mM to 110 mM (Fig. S5†), indicating that a minor change of solution resistance has little effect on ECL intensities. There is a linear relationship between the ECL intensity and the concentration of H_2_O_2_ from 1.0 to 100 μM, and the linear equation is *I* = 58.42 + 10.68*c* (μM) (*r* = 0.9996). The LOD is 0.27 μM at a signal-to-noise ratio of 3. Compared with other H_2_O_2_ ECL detection methods,^31^–^34^ the present method has similar sensitivity and is simpler and more cost-effective.

### SEESs for multiplex ECL analysis

**Fig. 3:**
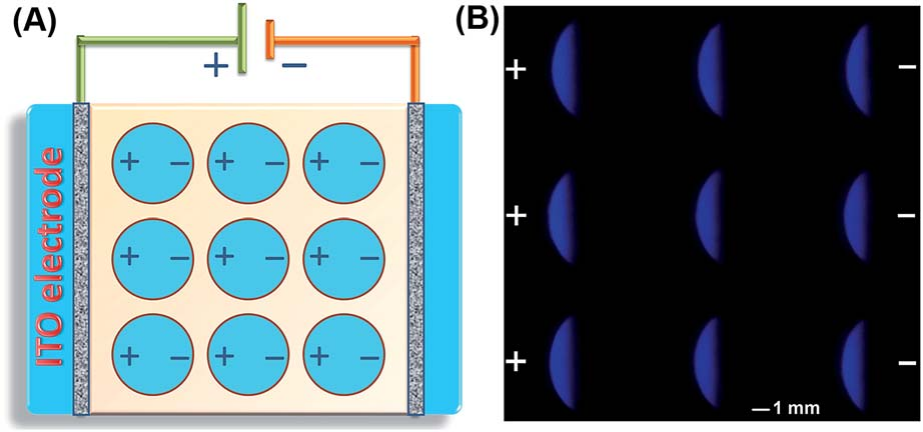
(A) Schematic diagram of the SEES with nine microelectrochemical cells from the top view. (B) ECL emission images of the luminol–H_2_O_2_ system using the SEES with nine microelectrochemical cells. 0.1 M carbonate buffer, pH 11.0; *c* (luminol): 1.0 mM; *c* (H_2_O_2_): 0.1 mM; the voltage applied to the SEES is 10 V.

The SEES can be used not only for single measurements, but also for multiplex measurements and high throughput measurements by simply covering the electrode with a waterproof PET label having a corresponding number and arrangement of holes. As a proof of concept, a PET label with nine circular holes was attached to a piece of ITO to make a SEES with nine microelectrochemical cells for the simultaneous measurement of nine samples (Fig. 3A). Fig. 3B shows the ECL emission images of the luminol–H_2_O_2_ system taken using a smartphone at the same concentrations by the SEES with nine microelectrochemical cells. The ECL emission images are clearly observed for all nine microelectrochemical cells and show the same intensity with a relative standard deviation of 2.7%, indicating satisfactory reproducibility. A SEES with eighty (8 × 10) microelectrochemical cells was also fabricated. In order to make it work well at lower and safer applied voltages, the gap distance between the cells was decreased from 1.0 mm (in the SEES with a single cell and nine cells) to 0.5 mm. Fig. S6† demonstrates the intense ECL image of the SEES with eighty (8 × 10) microelectrochemical cells with a relative standard deviation of 4.1% at an applied voltage of only 15 V which is much lower than the safe voltage. This indicates that SEESs are promising safe systems for multiplex measurements.

**Fig. 4:**
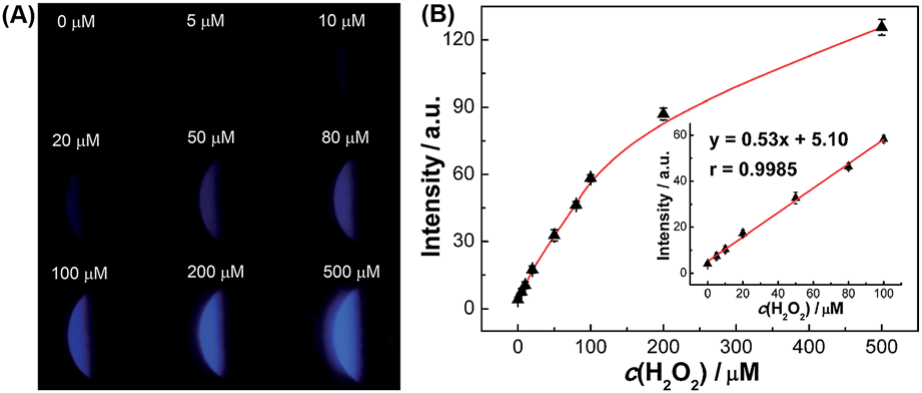
(A) ECL emission images of the luminol/H_2_O_2_ system with different concentrations of H_2_O_2_ using the SEES containing nine microelectrochemical cells. (B) Visualized quantitative detection of H_2_O_2_. 0.1 M carbonate buffer, pH 11.0; *c* (luminol): 1.0 mM; the voltage applied to the SEES is 10 V.

**Fig. 5:**
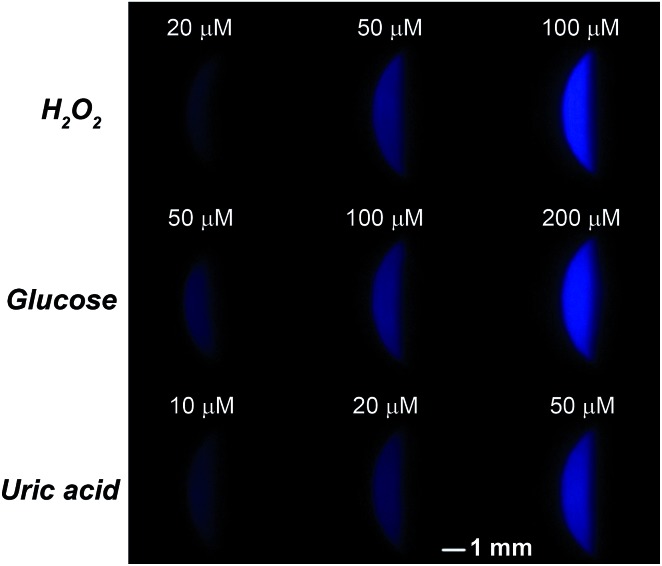
Multiplex ECL analysis of H_2_O_2_, glucose and uric acid. 0.1 M carbonate buffer, pH 11.0; *c* (luminol): 1.0 mM; the voltage applied to the SEES is 10 V.

To better demonstrate the performance of SEESs in multiplex analysis, hydrogen peroxide (H_2_O_2_), glucose and uric acid (UA) were used as model target analytes. Glucose and uric acid are important analytes and they can be indirectly monitored by ECL detection of H_2_O_2_ generated from enzymatic reactions using glucose oxidase and uricase, respectively. Firstly, the SEES with nine microelectrochemical cells was employed for the respective detection of H_2_O_2_, glucose and uric acid to establish the corresponding working curves. Fig. 4A shows the ECL emission images of the luminol–H_2_O_2_ system taken using a smartphone at different concentrations of H_2_O_2_. As the concentrations of H_2_O_2_ increased, the ECL signal became stronger. Fig. 4B shows that the ECL intensities increased linearly with the concentrations of H_2_O_2_ from 5.0 to 100.0 μM with a correlation coefficient of 0.9985. The linear equation is *I* = 5.10 + 0.53*c* (μM). A similar analysis of glucose and uric acid is shown in Fig. S7 and S8.† The linear relationship between the ECL intensity and the concentration of glucose is *I* = 2.27 + 0.27*c* (μM) with a correlation coefficient of 0.9940, ranging from 5.0 to 200.0 μM. The ECL intensities increased linearly with the concentrations of uric acid from 5.0 to 50.0 μM with a correlation coefficient of 0.9939. The linear equation is *I* = 10.26 + 0.86*c* (μM). Then, testing solutions containing different concentrations of H_2_O_2_, glucose and uric acid were added into different cells in the same SEES. Fig. 5 shows that the SEES allows the detection of different concentrations of multiple analytes simultaneously, demonstrating the feasibility of SEESs for multiplex analysis.

## Conclusions

In summary, a convenient electrochemical system, SEES, has been developed. The SEES enables a sensitive multiplex analysis of hydrogen peroxide, glucose and uric acid simultaneously using only one electrode at a safe voltage. In comparison with conventional electrochemical systems and bipolar electrochemical systems, SEESs are much simpler and cheaper for high throughput analysis since they use only one electrode and do not involve complex and costly fabrication of electrode arrays and electrode connectors. Moreover, SEES is free from the ECL background problem from driving electrodes in bipolar electrochemical systems. Besides ITO, numerous electrodes having resistance, such as conducting polymer electrodes, semiconductor electrodes, and carbon film electrodes (*e.g.* graphene electrodes, CNT electrodes, boron-doped diamond electrodes, and diamond-like carbon film electrodes), can be used to fabricate SEESs. In view of their favorable simplicity and versatility, SEESs will have broad applications.

## Experimental

### Chemicals and apparatus

Luminol was purchased from TCI (Shanghai, China). Hydrogen peroxide (H_2_O_2_), sodium bicarbonate (NaHCO_3_) and sodium hydroxide (NaOH) were supplied by Beijing Chemical Reagent Company (Beijing, China). Glucose and uric acid were bought from Sangon Biotech Co., Ltd. (Shanghai, China). Glucose oxidase was bought from Sigma-Aldrich Co. (St Louis, USA). Uricase was obtained from J&K Scientific Ltd. (Beijing, China). Triton X-100 was bought from Sinopharm Chemical Reagent Co. Ltd. (Shanghai, China). Indium tin oxide (ITO) conductive glass and waterproof self-adhesive polyethylene terephthalate (PET) label were ordered from Foshan City Meijingyuan Glass Co. Ltd and Jinfuzhou Paper Co. Ltd, respectively. Conductive carbon ink CH-8 was purchased from Jujo Printing Supplies & Technology Co. Ltd., China. A luminol stock solution of 10.0 mM was prepared by dissolving a certain amount of luminol in 0.1 M NaOH solution. Carbonate buffer solution (0.1 M, pH 11.0) was prepared using NaHCO_3_ and NaOH and was used in all ECL experiments. All the reagents were used without further purification. Deionized distilled water was used to prepare solutions.

Electrochemical potentials were supplied by a CHI 660C electrochemical workstation (Shanghai CHI Instruments Company, China) or a digitally regulated DC power supply (CE0120010T, Shanghai Kalaifei Company, China). ECL intensities were recorded using a BPCL ultraweak luminescence analyzer (Institute of Biophysics, Chinese Academy of Sciences). Visual detections were carried out in a dark box. The images of the ECL emission were captured using a Nubia Z7 max smartphone.

### Construction of SEES

The SEES was constructed by attaching a waterproof self-adhesive polyethylene terephthalate (PET) label with a rectangular hole (1.0 cm × 0.8 cm × 0.02 cm) onto a piece of ITO conductive glass (2.0 cm × 1.0 cm × 0.1 cm) and then connecting two copper wires to both ends of the insulating self-adhesive plastic film using conductive carbon ink. The rectangular hole on the PET label was easily punched using a puncher. For SEESs with multiple microelectrochemical cells, the holes on the PET label were fabricated using a Shangke Model H1380 cutting plotter from Jinan Shangke Trade and Business Co. Ltd. For the SEES with nine microelectrochemical cells, a waterproof self-adhesive PET label with nine circular holes (0.8 cm in diameter) with a gap distance of 0.1 cm was attached to a piece of ITO conductive glass (4.0 cm × 4.0 cm × 0.1 cm), and then two copper wires were connected onto both ends of the insulating self-adhesive plastic film using conductive carbon ink. For the SEES with eighty microelectrochemical cells, a waterproof self-adhesive PET label with eighty (8 × 10) circular holes (0.65 cm in diameter) with a gap distance of 0.05 cm was attached to a piece of ITO conductive glass (10.0 cm × 10.0 cm × 0.1 cm), and then two copper wires were connected onto both ends of the insulating self-adhesive plastic film using conductive carbon ink. The width of PET between the copper wire and holes was 0.2 cm.

### Procedure of luminol detection using the SEES with a single microelectrochemical cell

A volume of 7 μL of 0.1 M carbonate buffer solution (pH 11.0) containing 1.0 mM H_2_O_2_, 1% (w/w) Triton X-100 and different concentrations of luminol was pipetted into the microelectrochemical cell and used for ECL detection. Electrochemical potentials were supplied by a CHI 660C electrochemical workstation. ECL intensities were captured using a BPCL ultraweak luminescence analyzer with a voltage of 700 V applied to the photomultiplier tube.

### Procedure of hydrogen peroxide detection using the SEES with a single microelectrochemical cell

A volume of 7 μL of 0.1 M carbonate buffer solution (pH 11.0) containing 10 μM luminol, 1% (w/w) Triton X-100 and different concentrations of hydrogen peroxide was pipetted into the microelectrochemical cell and used for ECL detection. Electrochemical potentials were supplied by a CHI 660C electrochemical workstation. The ECL intensities were recorded using a BPCL ultraweak luminescence analyzer with a voltage of 600 V applied to the photomultiplier tube.

### Visual detection of hydrogen peroxide using SEESs with multiple microelectrochemical cells

A volume of 5 μL of 0.1 M carbonate buffer solution (pH 11.0) containing 1.0 mM luminol, 1% (w/w) Triton X-100 and different concentrations of hydrogen peroxide was pipetted into different microelectrochemical cells. Electrochemical potentials were supplied by a digitally regulated DC power supply. The ECL emission images were captured using a Nubia Z7 max smartphone with an exposure time of 3 seconds. The light spots on the pictures were analyzed using ImageJ software.

### Visual detection of glucose and uric acid using SEESs with multiple microelectrochemical cells

A different concentration of glucose and uric acid was mixed with 100 μg mL^–1^ of glucose oxidase and uricase, respectively. The mixtures were mixed and reacted at room temperature for 10 min to produce H_2_O_2_. Then, a volume of 5 μL of 0.1 M carbonate buffer solution (pH 11.0) containing 1.0 mM luminol, 1% (w/w) Triton X-100 and the mixture was pipetted into different microelectrochemical cells. Electrochemical potentials were supplied by a digitally regulated DC power supply. The ECL emission images were captured using a Nubia Z7 max smartphone with an exposure time of 3 seconds. The light spots on the pictures were analyzed using ImageJ software.

## Conflicts of interest

There are no conflicts to declare.

## Supplementary Material

Supplementary information
