# Supplementary material for: A single-electrode electrochemical system for multiplex electrochemiluminescence analysis based on a resistance induced potential difference
Source: Chem Sci. 2018 Mar 19;9(16):3911–6. doi: 10.1039/c8sc00410b (PMC5935220; doi:10.1039/c8sc00410b)
Supplement: Supplementary file 1 [file SC-009-C8SC00410B-s001.pdf]

Electronic Supplementary Material (ESI) for Chemical Science.
This journal is © The Royal Society of Chemistry 2018

Supplementary Information

Single-electrode electrochemical system for multiplex electrochemiluminescent analysis based on resistance induced potential difference

Wenyue Gao,[a,b] Kateryna Muzyka,[a,c] Xiangui Ma,[a,b] Baohua Lou[a] and Guobao Xu*[a]

[a] State Key Laboratory of Electroanalytical Chemistry, Changchun Institute of Applied Chemistry, Chinese Academy of Sciences, Changchun, Jilin 130022, P.R. China.

[b] University of Chinese Academy of Sciences, Beijing 100049, P.R. China.

[c] Laboratory of Analytical Optochemotronics, Department of Biomedical Engineering, Kharkiv National University of Radio Electronics, Kharkiv 61166, Ukraine.

Table of Contents

Fig. S1 Current-time curves on the SEES in the absence and presence of electrolyte.

Fig. S2 Effect of ITO resistance on the ECL intensity.

Fig. S3 Dependence of the ECL intensities on the concentrations of luminol.

Fig. S4 ECL detection toward hydrogen peroxide using SEES having one microelectrochemical cell.

Fig. S5 Dependence of the ECL intensities on the concentrations of buffer solution.

Fig. S6 Performance of the SEES with eighty microelectrochemical cells.

Fig. S7 ECL analysis of glucose using SEES containing nine microelectrochemical cells.

Fig. S8 ECL analysis of uric acid using SEES containing nine microelectrochemical cells.

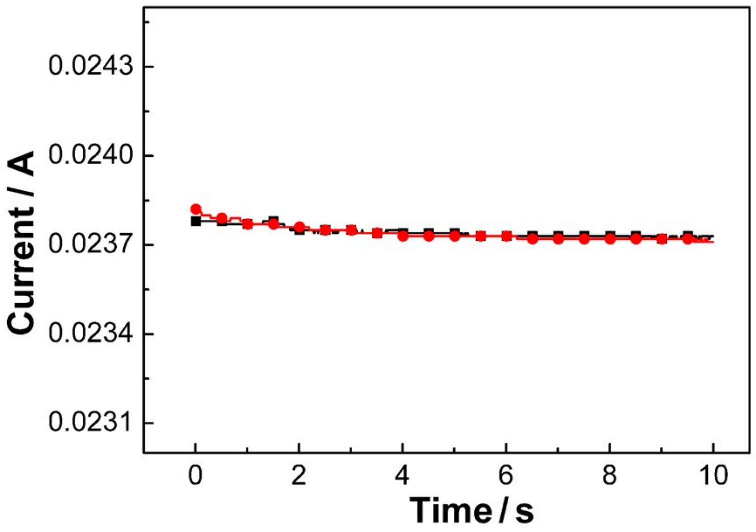

Fig. S1 Current-time curves on the SEES in the absence of any solution (black line), and in the presence of 0.1 M carbonate buffer (pH 11.0) containing 10 μM of luminol and 0.1 mM of H_2O_2 (red line). The voltage applied to the SEES is 2.0 V.

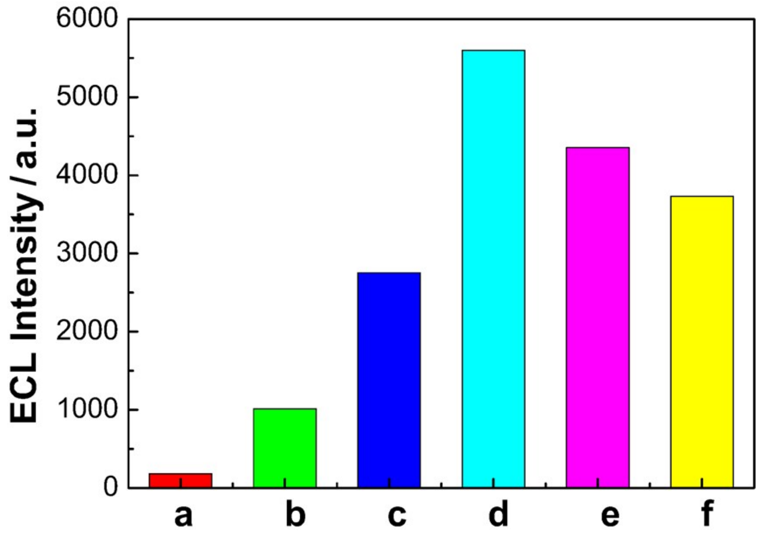

Fig. S2 Effect of ITO resistance on the ECL intensity. The column from a to f represents different ITO resistance (Ohms/square): 10, 20, 50, 100, 200, 500. 0.1 M, pH 11.0 carbonate buffer solution (CBS); *c*(luminol): 10 μM; *c*(H_2O_2): 1.0 mM; PMT: 650V; the voltage applied to the SEES is 2.0 V.

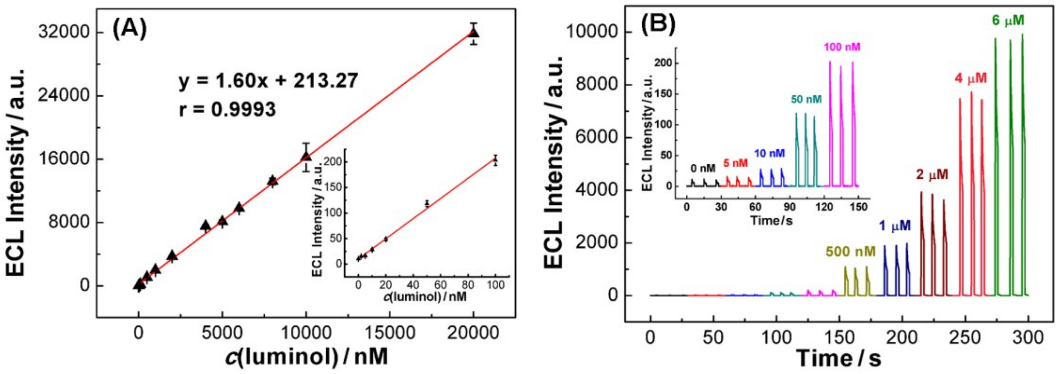

Fig. S3 Dependence of the ECL intensities on the concentrations of luminol. (A) Linear calibration curve of luminol. (B) ECL profiles with three consecutive measurements at different concentrations of luminol. Inset is the enlarged one from 0 to 100 nM. 0.1 M carbonate buffer, pH 11.0; $c(H_2O_2)$: 1.0 mM; PMT: 700V; the voltage applied to the SEES is 2.0 V.

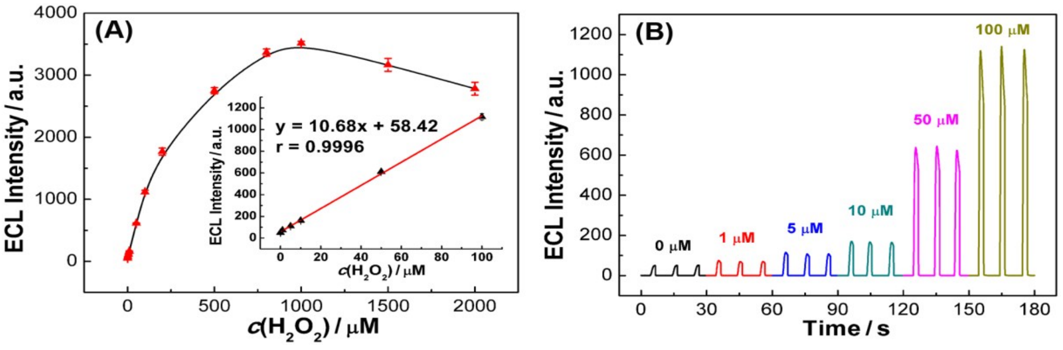

Fig. S4 ECL detection toward hydrogen peroxide using SEES having one microelectrochemical cell. (A) Dependence of ECL intensity on the concentrations of H_2O_2. Inset: Linear calibration curve of H_2O_2 from 1.0 to 100 μM. (B) ECL profiles with three consecutive measurements at different concentrations of H_2O_2. 0.1 M carbonate buffer, pH 11.0; c(luminol): 10 μM; PMT: 600V; the voltage applied to the SEES is 2.0 V.

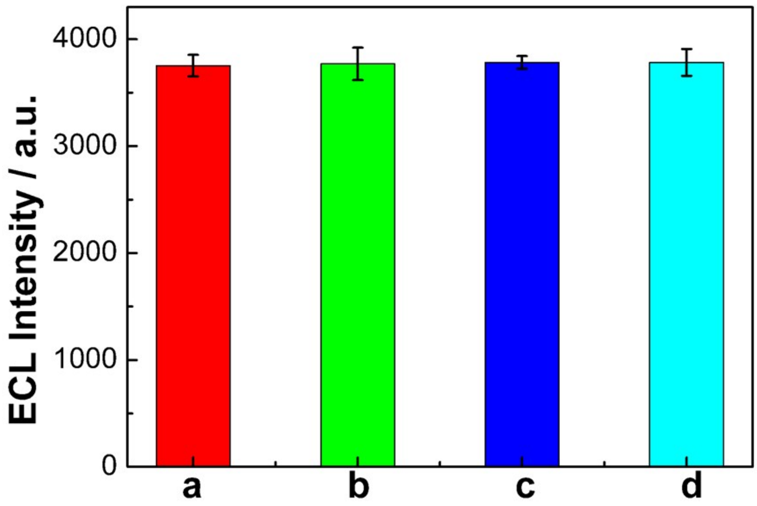

Fig. S5 Dependence of the ECL intensities on the concentrations of carbonate buffer solution (pH 11.0): a, 50 mM; b, 100 mM; c, 101 mM; d, 110 mM. c(luminol): 10 μM; $c(H_2O_2)$: 0.1 mM; PMT: 700V; the voltage applied to the SEES is 2.0 V.

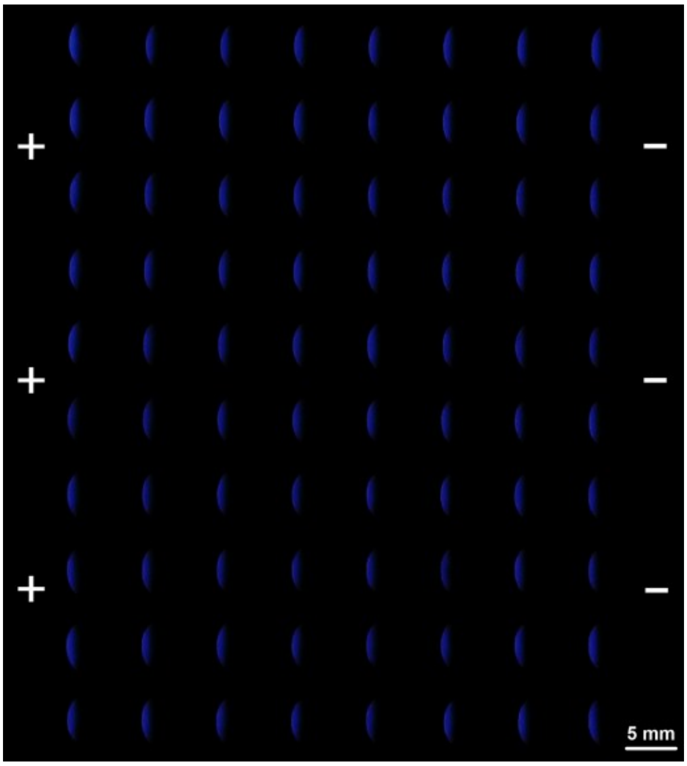

Fig. S6 Performance of the SEES with eighty microelectrochemical cells. ECL emission images of luminol/H_2O_2 system using SEES with eighty (8×10) microelectrochemical cells. 0.1 M carbonate buffer, pH 11.0; c(luminol): 1.0 mM; $c(H_2O_2)$: 0.1 mM; the voltage applied to the SEES is 15 V.

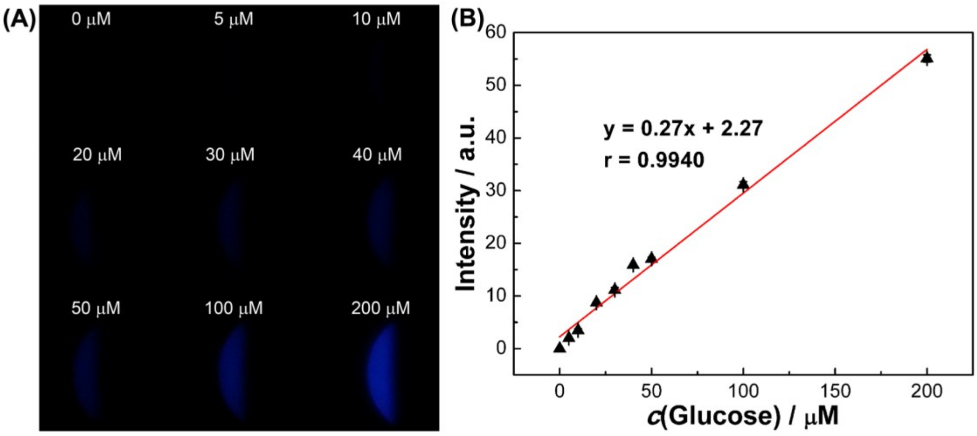

Fig. S7 ECL analysis of glucose using SEES containing nine microelectrochemical cells. (A) ECL emission images of luminol/glucose oxidase/glucose system with different concentrations of glucose using SEES containing nine microelectrochemical cells. (B) Visualized quantitative detection of glucose. 0.1 M carbonate buffer, pH 11.0; *c*(luminol): 1.0 mM; the voltage applied to the SEES is 10 V.

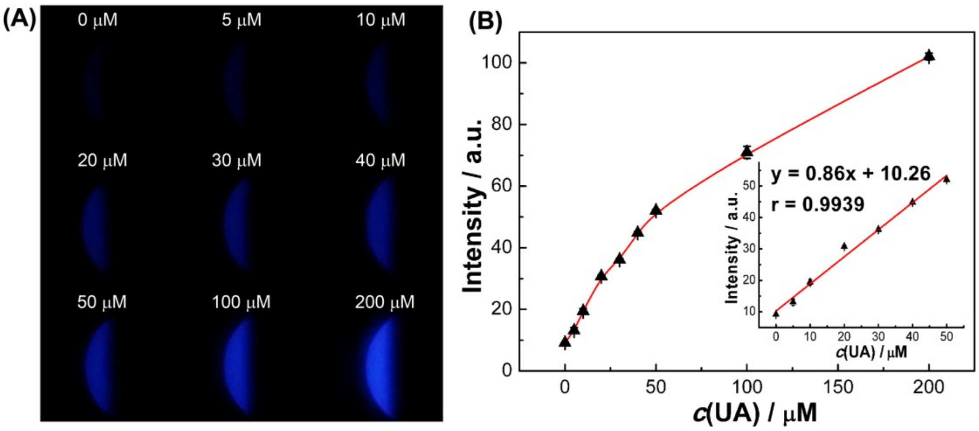

Fig. S8 ECL analysis of uric acid using SEES containing nine microelectrochemical cells. (A) ECL emission images of luminol/uricase/uric acid system with different concentrations of uric acid (UA) using SEES containing nine microelectrochemical cells. (B) Visualized quantitative detection of uric acid. 0.1 M carbonate buffer, pH 11.0; *c*(luminol): 1.0 mM; the voltage applied to the SEES is 10 V.
